# Comorbidities in women with polycystic ovary syndrome: a sibling study

**DOI:** 10.1186/s12905-024-03028-9

**Published:** 2024-04-05

**Authors:** Beata Vivien Boldis, Ilona Grünberger, Agneta Cederström, Jonas Björk, Anton Nilsson, Jonas Helgertz

**Affiliations:** 1 Department of Public Health Sciences, Stockholm University, Albanovägen 12, Hus 4, plan 5, 106 91 Stockholm, Sweden; 2 Epidemiology, Population Studies and Infrastructures (EPI@LUND), Lund University, Lund, Sweden; 3 Department of Laboratory Medicine, Lund University, 22100 Lund, Sweden; 4 Centre for Economic Demography, School of Economics and Management, Lund University, 22100 Lund, Sweden; 5 Department of Economic History, Lund University, 22100 Lund, Sweden; 6 Institute for Social Research and Data Innovation, Minnesota Population Center, University of Minnesota, Minneapolis, MN 55455 USA

**Keywords:** Polycystic ovary syndrome, Comorbidity, Sibling fixed effect

## Abstract

**Background:**

Polycystic ovary syndrome (PCOS) has previously been associated with several comorbidities that may have shared genetic, epigenetic, developmental or environmental origins. PCOS may be influenced by prenatal androgen excess, poor intrauterine or childhood environmental factors, childhood obesity and learned health risk behaviors. We analyzed the association between PCOS and several relevant comorbidities while adjusting for early-life biological and socioeconomic conditions, also investigating the extent to which the association is affected by familial risk factors.

**Methods:**

This total-population register-based cohort study included 333,999 full sisters, born between 1962 and 1980. PCOS and comorbidity diagnoses were measured at age 17-45 years through national hospital register data from 1997 to 2011, and complemented with information on the study subjects´ early-life and social characteristics. In the main analysis, sister fixed effects (FE) models were used to control for all time-invariant factors that are shared among sisters, thereby testing whether the association between PCOS and examined comorbidities is influenced by unobserved familial environmental, social or genetic factors.

**Results:**

Three thousand five hundred seventy women in the Sister sample were diagnosed with PCOS, of whom 14% had obesity, 8% had depression, 7% had anxiety and 4% experienced sleeping, sexual and eating disorders (SSE). Having PCOS increased the odds of obesity nearly 6-fold (adjusted OR (aOR): 5.9 [95% CI:5.4-6.5]). This association was attenuated in models accounting for unobserved characteristics shared between full sisters, but remained considerable in size (Sister FE: aOR: 4.5 [95% CI: 3.6-5.6]). For depression (Sister FE: aOR: 1.4 [95% CI: 1.2-1.8]) and anxiety (Sister FE: aOR: 1.5 [95% CI: 1.2-1.8), there was a small decrease in the aORs when controlling for factors shared between sisters. Being diagnosed with SSE disorders yielded a 2.4 aOR (95% CI:2.0-2.6) when controlling for a comprehensive set of individual-level confounders, which only decreased slightly when controlling for factors at the family level such as shared genes or parenting style. Accounting for differences between sisters in observed early-life circumstances influenced the estimated associations marginally.

**Conclusion:**

Having been diagnosed with PCOS is associated with a markedly increased risk of obesity and sleeping, sexual and eating disorders, also after accounting for factors shared between sisters and early-life conditions.

**Supplementary Information:**

The online version contains supplementary material available at 10.1186/s12905-024-03028-9.

## Introduction

Polycystic ovary syndrome (PCOS), a subtype of ovarian dysfunction (OD), is a heterogenous disorder that has a prevalence of 4-20% among premenopausal women, depending on the criteria used for diagnosis [1–3]. Despite PCOS showing a similar prevalence to diabetes mellitus (DM) [4], it still remains one of the most poorly understood, undertreated and misclassified syndromes with long-term health consequences [5–7].

PCOS is associated with numerous comorbidities, including obesity, depression, anxiety, sleep apnea and eating and sexual disorders [8–12]. Obesity, metabolic disorders and insulin resistance are often observed among women with the most severe phenotypes of PCOS [13, 14]. Excess androgen among women with PCOS facilitates abdominal and visceral adiposity, which is commonly seen among patients with insulin resistance [15, 16]. This surplus of androgen may also lead to psychiatric disorders such as depressive disorder and generalized anxiety disorder [17].

Previous studies have reported a higher risk for eating disorder among women with PCOS [18, 19], supporting the idea that hyperandrogenism can intensify food cravings, over-eating and bulimic behavior [20, 21]. There may also be an association between obstructive sleep apnea (OSA) and PCOS, since OSA is also associated with obesity and depressive disorders. Since the endocrine system has an important role in the regulation of comorbidities characterized by metabolic disturbances, as well as PCOS, it is likely that these conditions cluster together. This suggests a complex relationship between the different conditions [22]. OSA is also associated with depression, which could potentially cause a lower quality of life for people with PCOS [20, 23]. Finally, sexual dysfunction among women has been linked to obesity, depression and anxiety. Due to these co-existing comorbidities, lower sexual satisfaction may occur among PCOS patients [9, 24, 25]. Still, PCOS screening among women with these comorbidities is not fully stipulated by existing guidelines [26], possibly resulting in undiagnosed cases and cumulative negative health outcomes [27]. The first aim of this study is to investigate the association between PCOS and several comorbidities; obesity, depression, anxiety and sleeping, sexual and eating disorders (SSE).

The etiology of PCOS is still ambiguous with a range of conceivable genetic and environmental contributing factors [28, 29]. Women have a higher risk of developing PCOS when having a first-degree female relative with the syndrome [30, 31], and one third of sisters of women with PCOS meet the diagnostic criteria of the syndrome as well [30]. In our prior research, we found that the risk for PCOS increased almost 3-fold when the mother, and by nearly 5-fold when a sister, had already been diagnosed with PCOS [32]. Further evidence suggests that male first-degree relatives of PCOS women have similar endocrine and metabolic risks [33], co-occurring with typical phenotype of male-patterned baldness [33]. Therefore, building on our earlier research finding familial clustering of the trait [32], the second and main aim of this present study is to explore the extent to which the associations between PCOS and examined comorbidities is affected by familial confounding. This is done through a sister fixed effects (FE) approach, exploiting variation between full sisters, cancelling out the influence of time-invariant factors – observed and unobserved – that are shared between sisters.

PCOS has been suggested to be influenced by intrauterine development [28, 34, 35]. In particular, it has been suggested that hyperandrogenic fetal ovaries might reprogram developmental processes that can lead to adult PCOS phenotype [34]. As outlined in our previous publication [32], we found evidence for the influence of early-life factors such as gestational age and one-minute Apgar score. Therefore, to further elaborate on our previous findings, the third aim of our study is to quantify the association between PCOS and examined comorbidities when controlling for differences in early-life conditions between full sisters.

## Methods

### Study population

This study was part of the Swedish Interdisciplinary Panel (SIP) project with an individual-level database, administered at the Centre for Economic Demography, Lund University. Through the unique Swedish personal identification number assigned at birth or immigration, several national registers, such as the Swedish Medical Birth Register (MBR), the Swedish National Patient Register (NPR), Total Population Register (TPR), Register of Participation in Education (UREG) and the Multi-Generational Register have been linked together, allowing for a uniquely detailed and longitudinal description of the health and socioeconomic characteristics of the population. We extracted a population consisting of all women born between 1962 and 1980 (*N* = 1,352,019), linking them to their biological parents and siblings.

**Fig. 1:**
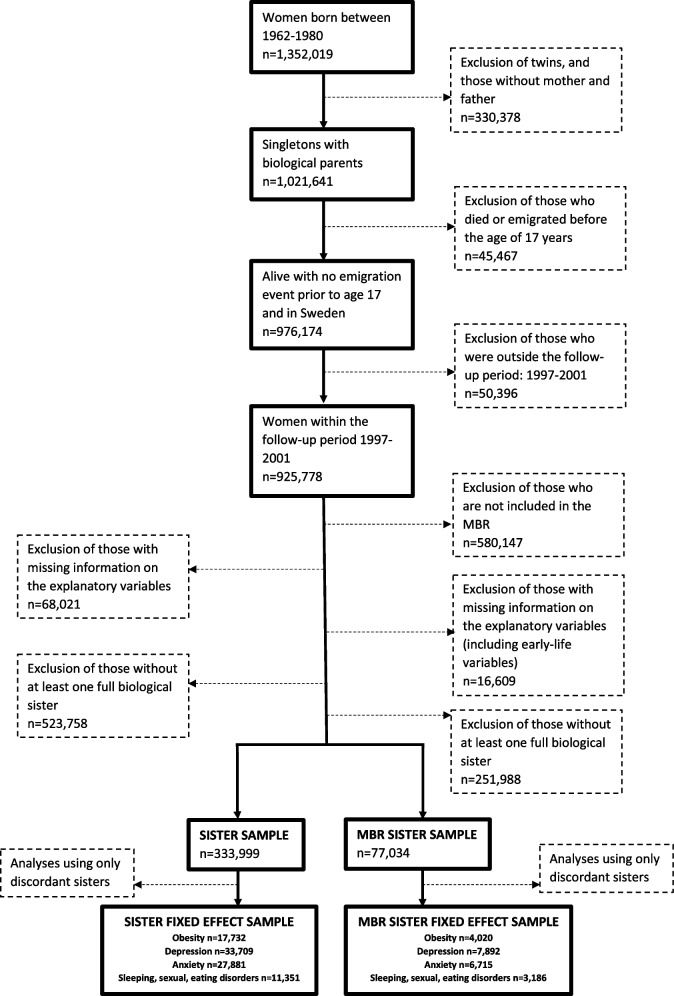
Flow diagram of the study sample creation

Multiple births and women with a missing link to either biological parent were excluded (*n*=330, 378). We further excluded those who died or emigrated before the age of 17 or before the start of the follow-up period (*n* = 45,467), and those who were outside of the follow-up period (1997-2011) (*n* = 50,396). After excluding those with missing information on the explanatory variables and those without at least one full biological sister, two sub-samples were created. One consisted of women with at least one full sister (Sister sample: *n* = 333,999) and another with additional restriction to women with information on characteristics measured at birth, retrieved from the MBR, implying only Swedish-born women born between 1973-1980 are included (MBR Sister sample: *n* = 77,034). The flow chart in Fig. 1 illustrates how the analytical samples were generated.

**Fig. 2:**
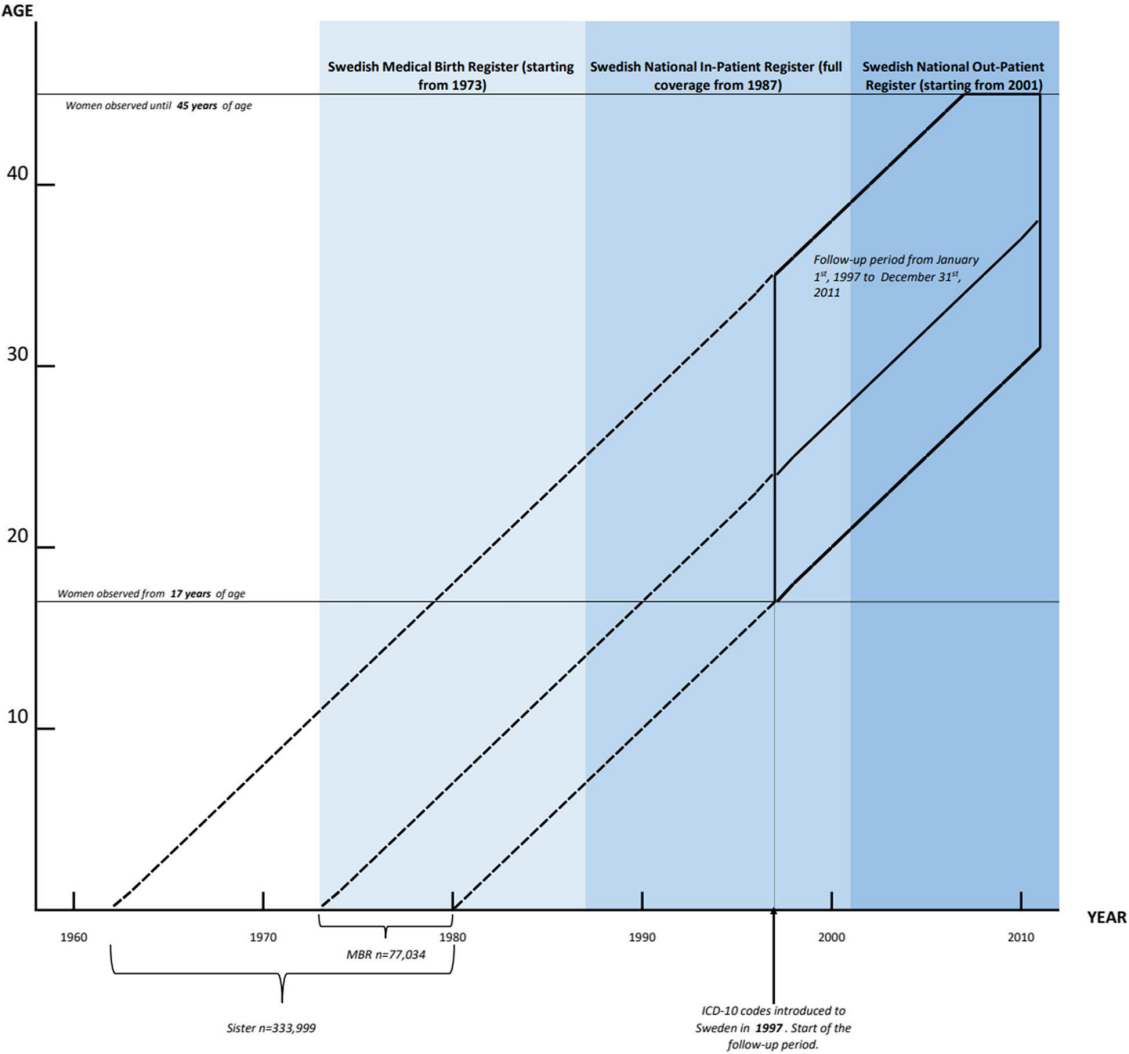
Data availability from Swedish national registers and sampling windows

The follow-up period starts on January 1, 1997, as International Classification of Disease (ICD) 10 codes started being used in the same year in Sweden, with the sample restricted to women observed between the ages of 17 and 45 years. The study population was followed continuously until whichever happened first: turning 45 years of age, death, emigration, or the end of our follow-up period on December 31, 2011. Figure 2 depicts the samples used in different parts of analysis and also illustrates the periods of availability of relevant data in Swedish administrative registers.

### Outcome: comorbidity variables

Using the NPR, we defined the following potential comorbidities, using the ICD-10 diagnostic codes: obesity (E66), depression (F32 – Depressive episode, and F33 – Recurrent depressive disorder), anxiety (F41 – Other anxiety disorder), and SSE disorders (F51 – Non-organic sleeping disorder, F52 – Sexual dysfunction not caused by organic dysfunction or disease, F50 – Eating disorders). Each condition was defined as a binary variable, indicating whether the individual had been diagnosed at any time during the follow-up period and not necessarily subsequent to the PCOS diagnosis.

### Exposure: PCOS and exclusion criteria

PCOS was identified through the NPR [36] at any time during the follow-up period as the binary exposure, including both inpatient and outpatient diagnoses (E28), with the vast majority of the diagnoses coming from outpatient data (99.7%). The aggregated ICD-10 code E28 was used, due to limited access to the more detailed PCOS diagnosis code (ICD-10: E28.2). Women diagnosed with conditions that could cause similar symptoms to PCOS, including Turner syndrome (Q96), Malignant neoplasm of ovary (C56), Suprarenal tumor (C74), Adrenogenital syndrome (E25), Cushing disease (E24) and Pituitary hypersecretion (E22), have been excluded to ensure specificity, similar to previous literature [37]. Both main and contributing diagnoses were considered. In our main analysis, we only included women who were never diagnosed with any of the exclusion criteria, regardless of whether these happened before or after the PCOS diagnosis.

### Covariates

#### Family characteristics

Highest maternal and paternal education attainment were obtained from UREG and categorized as primary/secondary or university, and were used as proxies for their daughters’ socioeconomic status in childhood. Mother’s, father’s and offspring’s country of birth was obtained from the TPR and grouped as a three-level variable to Sweden; Europe, North America and Oceania; and Africa, Asia and South America. Birth order was created from maternal live birth and grouped as first born, second born and third born or higher. Mother’s age at birth was grouped into less than or equal to 18 years of age, between 19 and 35 years of age and greater than or equal to 36 years of age.

#### Adult characteristics

The index individual’s highest attained education was obtained from UREG and categorized as Primary/Secondary or University. Civil status was extracted from the TPR and used to distinguish between married/in a registered partnership or not married/not in a registered partnership, using the civil status of the highest observed age between the ages of 17 to 45 years. Additionally, the final models were adjusted for the index woman’s residence at the time of the follow-up, where residence status was grouped into today’s 21 counties.

#### Early-life factors

Birthweight, one-minute Apgar score and gestational age were extracted from the MBR. Birthweight was categorized into 500-g groups (≤2,499g, 2,500-2,999g, 3,000g-3,499g, 3,500-3,999g, 4,000-4,499g and ≥4,500g). Gestational age was measured in completed weeks of gestation and grouped as extremely preterm (<28 weeks), very preterm (28-32 weeks), moderate to late preterm (33-36 weeks), full-term (37-41 weeks) and post-term birth (≥42 weeks). Gestational age was based on self-reported first dates of the last menstrual period or on results of ultrasound examinations. One-minute Apgar score was categorized as 10, 9, 8 and less than or equal to 7, as a standardized assessment of health signs of newborns immediately after birth [38].

### Statistical analysis

Statistical analyses were performed using STATA/MP 17.0 (StataCorp). The main analyses were restricted to individuals with non-missing information on all covariates included in the models.

In order to address the first aim of the study, we treated the sisters as unrelated individuals and thus used unconditional logistic regression to examine the association between PCOS and each comorbidity condition in the Sister sample, while controlling for previously outlined covariates. Covariates were added to the model group-wise, from controls for year of birth to family background characteristics and adult characteristics. Additionally, the same models were run on a sample that was not restricted to full sisters.

Addressing the second aim, comparing the associations between PCOS and examined comorbidity conditions among full sisters, a FE logistic regression was used (also known as conditional logistic regression). This accounts for unobserved, time-invariant factors coming from the shared family environmental, social or genetic factors that could affect both PCOS and the selected comorbidity.

A potential downside to this approach pertains to it requiring within-sister combination variation in the outcome. Thus, at least one (but not every) sister has to be diagnosed with the examined comorbidity, meaning that only discordant sisters are included in the analysis. This restriction reduced the sample for the examination of all outcomes; obesity (*n* = 7,994 families, *n* = 17,732 women), depression (*n* = 15,232 families, *n* = 33,709 women), anxiety (*n* = 12,597 families, *n* = 27,881 women), SSE disorders (*n* = 5,129 families, *n* = 11,351 women).

For each selected comorbidity, we compared the results from unconditional logistic regression models and sister FE models on the Sister and MBR Sister sample, respectively. The FE approach adjusts for unmeasured factors that are shared between the sisters, such as genes which may cause a predisposition to disease or other factors linked to sharing an environment during the upbringing, such as parenting style, and attitudes towards exercise and diet. While we are unable to quantify the relative importance of each of the aforementioned factors, the method allows for obtaining the association after adjusting for shared factors between sisters.

To investigate our third aim, to what extent the association between PCOS and the selected comorbidities changes when controlling for early-life factors such as birthweight, gestational age and one-minute Apgar score, we restricted our sample to full sisters born in Sweden between 1973-1980 and with information on their selected birth characteristics available (MBR Sister sample).

### Sensitivity analysis

Due to discordancy in the outcome variable and also the fact that each PCOS diagnosed woman needed to have a sister, there is a risk that the sample is selected and thereby yielding results with limited external validity in a larger representative sample. We address this concern by reporting results from models estimated on the sibling FE sample but without aforementioned FE. In addition, we compare these results to the corresponding results of a sample without restrictions to sisters. We argue that this provides a good approximation of the degree to which the sibling FE sample can be used to understand associations in a population of outcome-concordant siblings. Additionally, we estimate random intercept models on the Sister sample and Sister MBR sample. Random intercept models are less restrictive in terms of both study sample and the ability to obtain parameter estimates for independent variables that do not display any variation within sibling combinations. The disadvantage, however, is that the appropriateness of the method requires that there is no correlation between the random effects and the independent variables. We therefore used the Hausman (1978) [39] specification test to detect violations of the random effects approach assumptions.

The diagnostic codes for SSE disorders were combined, due to the generally low prevalence of these conditions (0.64%, 0.40% and 0.64% in the total sample, respectively), and were treated as one binary variable in the main analyses. Therefore, a separate analysis for each of the disorders was conducted.

## Results

### Descriptive characteristics

Descriptive characteristics of the study population are presented in Table 1 for two samples of women that we used in our analyses: one sample with women born in Sweden in 1962-1980 with at least one full sister (Sister sample, *n* = 333,999), and another sample of women born in Sweden in 1973-1980 with at least one full sister (MBR Sister sample, *n* = 77,034). Descriptive characteristics of additional sub-samples such as: without restriction to sisters (*n* = 857,757), concordant sisters without PCOS (*n* = 326,422), concordant sisters with PCOS (*n* = 137), and discordant sisters (*n* = 7,440), are shown in Appendix Table A.1.

**Table 1:** Descriptive characteristics of the study populations used in main analysis

	**SISTER** ***N*=333,999**	**MBR SISTER**^**a**^ ***N*=77,034**
**Polycystic ovary syndrome**	3,570 (1.1%)	1,212 (1.6%)
**Obesity**	9,096 (2.7%)	2,250 (2.9%)
**Depression**	17,264 (5.2%)	4,402 (5.7%)
**Anxiety**	14,017 (4.2%)	3,652 (4.7%)
**Sleeping, sexual and eating disorder**	5,304 (1.5%)	1,605 (2.1%)
**Mother´s educational attainment**		
*Primary, Secondary*	254,117 (76.1%)	51,858 (67.43%)
*University*	79,882 (23.9%)	25,176 (32.7%)
**Father´s educational attainment**		
*Primary, Secondary*	262,027 (78.5%)	55,635 (72.2%)
*University*	71,972 (21.6%)	21,399 (27.8%)
**Mother´s origin**		
*Sweden*	297,211 (89.0%)	71,200 (92.4%)
*Europe, North America and Oceania*	30,984 (9.3%)	5,426 (7.1%)
*Africa, Asia, South America*	5,804 (1.7%)	408 (0.5%)
**Father´s origin**		
*Sweden*	296,177 (88.7%)	70,804 (91.9%)
*Europe, North America and Oceania*	31,466 (9.4%)	5,543 (7.2%)
*Africa, Asia, South America*	6,356 (1.9%)	687 (0.9%)
**Origin**		
*Sweden*	321,347 (96.2%)	77,034(100%)
*Europe, North America and Oceania*	7,678 (2.3%)	-
*Africa, Asia, South America*	4,974 (1.5%)	-
**Birth order**		
*First born*	121,289 (36.3%)	29,116 (37.8%)
*Second born*	133,505 (40.0%)	34,394 (44.7%)
*Third born or higher*	79,205 (23.7%)	13,524 (17.5%)
**Mother´s age at index woman´s birth, years**		
*Less than or equal to 18*	9,722 (2.9%)	1,316 (1.7%)
*Between 19-35*	309,704 (92.7%)	73,283 (95.1%)
*Greater than 35*	14,573 (4.4%)	2,435 (3.2%)
**Educational attainment**		
*Primary, Secondary*	167,937 (50.3%)	31,230 (40.6%)
*University*	166,062 (49.7%)	45,804 (59.4%)
**Civil status**		
*Not married, not registered partnership*	169,598 (50.8%)	42,800 (55.6%)
*Married, registered partnership*	164,401 (49.2%)	34,234 (44.4%)

*Abbreviation*: *MBR* Medical Birth Registry

^a^Available for a subgroup of women born between 1973 and 1980

**Fig. 3:**
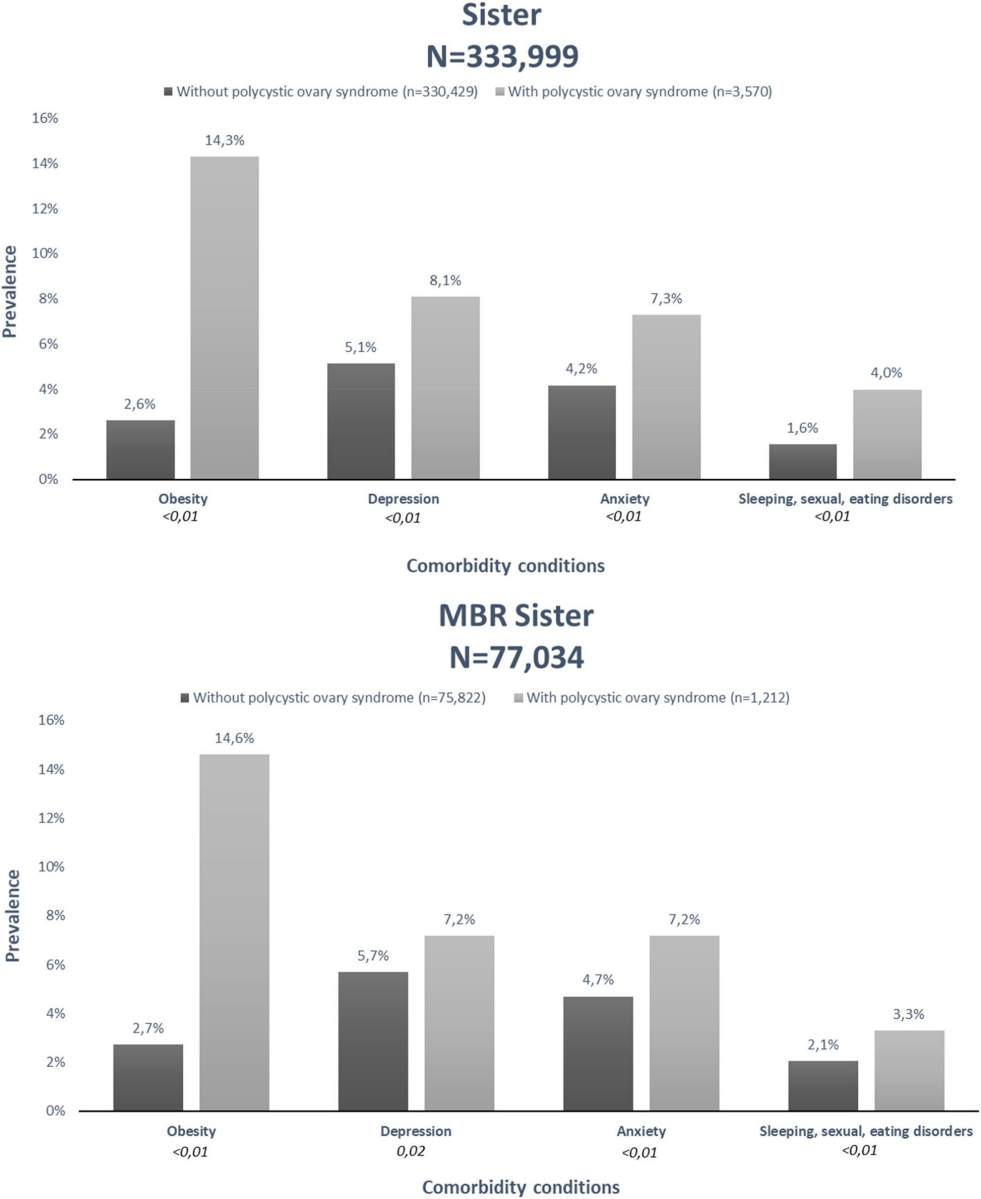
Prevalence of comorbidity conditions among women with and without PCOS

From a total of 333,999 women aged 17 to 45 years and followed from 1997-2011, 3,570 were diagnosed with PCOS, of which 14% were diagnosed with obesity, 8% with depression, 7% with anxiety and 4% with SSE disorders during follow-up. The prevalence of the comorbidities among cases of PCOS remained very similar in the MBR Sister sample (Fig. 3).

### Main analysis

#### Associations between PCOS and the selected comorbidities

The minimally adjusted odds ratio (mORs), only adjusted for birth year, associated with PCOS in Model 1 (Sister sample, Table 2) was 5.88 (95% CI: 5.34-6.47) for being diagnosed with obesity, 1.54 (95% CI: 1.37-1.74) for depression, 1.69 (95% CI: 1.49-1.93) for anxiety and 2.35 (95% CI: 1.98-2.79) for SSE disorders. Extending the models to account for other risk factors only changed the associations marginally, with adjusted ORs (aOR) of 5.92, 1.58, 1.72 and 2.36 in the fully adjusted Model 3. Results from otherwise identical models but not restricted to full sisters (Table A.2), confirm the robustness of these findings. Thus, restricting the sample to full sisters is not driving observed associations between PCOS and examined comorbidities, something that applies to both the mORs and the aORs.

**Table 2:** Results from logistic regression (odds ratios and 95% confidence intervals) comparing unconditional and fixed effects logit models predicting associations between polycystic ovary syndrome and comorbidities among women in Sweden, Sister sample

	**OBESITY**			**DEPRESSION**			**ANXIETY**			**SLEEPING, SEXUAL, EATING DISORDERS**		
	**M1**	**M2**	**M3**	**M1**	**M2**	**M3**	**M1**	**M2**	**M3**	**M1**	**M2**	**M3**
**Sister Unconditional**												
	***n*=333,999**			***n*=333,999**			***n*=333,999**			***n*=333,999**		
Polycystic ovary syndrome	5.882*** (5.341 - 6.478)	5.868*** (5.324 - 6.468)	5.918*** (5.362 - 6.531)	1.542*** (1.366 - 1.742)	1.528*** (1.353 - 1.726)	1.574*** (1.392 - 1.779)	1.694*** (1.491 - 1.925)	1.679*** (1.478 - 1.908)	1.720*** (1.512 - 1.956)	2.350*** (1.981 - 2.787)	2.340*** (1.973 - 2.776)	2.364*** (1.992 - 2.804)
**Sister FE**												
	***n*=17,732** **Sibling units=7,994**			***n*=33,709** **Sibling units=15,232**			***n*=27,881** **Sibling units=12,597**			***n*=11,351** **Sibling units=5,129**		
Polycystic ovary syndrome	4.460*** (3.596 - 5.532)	4.441*** (3.580 - 5.510)	4.474*** (3.598 - 5.562)	1.447*** (1.196 - 1.749)	1.446*** (1.196 - 1.749)	1.439*** (1.184 - 1.748)	1.417*** (1.161 - 1.730)	1.428*** (1.169 - 1.744)	1.467*** (1.197 - 1.798)	2.296*** (1.701 - 3.100)	2.298*** (1.702 - 3.103)	2.289*** (1.690 - 3.101)

*Abbreviations*: *FE* Fixed effect

M1: models adjusted for Birth year

M2: models adjusted for Birth year and Family background [Mother´s educational attainment, Father´s educational attainment, Mother´s origin, Father´s origin, Index women´s origin, Birth order, Mother´s age at index woman´s birth]

M3: models adjusted for Birth year, Family background [Mother´s educational attainment, Father´s educational attainment, Mother´s origin, Father´s origin, Index women´s origin, Birth order, Mother´s age at index woman´s birth] and Adult characteristics [Educational attainment, Civil status, County]

^***^p<0.001

^**^p<0.01

^*^p<0.05

#### The influence of familial risk factors

We investigated to what extent the association between being diagnosed with PCOS and each comorbidity can be attributed to factors shared between sisters. We present the results from sibling FE models in Table 2, with the corresponding results from random effect models presented in the Appendix (Table A.3), yielding similar conclusions. A strong positive association between PCOS and obesity was still observed in the sibling FE model (Sister FE Model 3: aOR: 4.47 [95% CI: 3.60-5.56]), although is attenuated compared to the results from the Sister model.

For depression (Sister FE Model 3: aOR: 1.44 [95% CI: 1.18-1.75]) and anxiety (Sister FE Model 3: aOR: 1.47 [95% CI: 1.20-1.80]), there was a small decrease in the aORs when also controlling for time-invariant factors shared between sisters. Being diagnosed with SSE disorders among women with PCOS yielded a 2.37 aOR in the Sister sample (Model 3: aOR: 2.37 [95% CI:1.99-2.80]), which decreased slightly when controlling for shared factors at the family level (Sister FE Model 3: aOR: 2.29 [95% CI: 1.69-3.10]).

#### Importance of early-life factors

The observed associations between PCOS and comorbidities where we also adjust for birthweight, gestational age and one-minute Apgar score are presented in Table 3. The estimates in Model 1 are rather similar to those obtained in the larger sister sample and in the without restriction to sisters sample (Appendix. Table A.2), showing that the minimally adjusted associations between PCOS and the examined outcomes are similar in the more restricted MBS Sister sample. The association between PCOS an obesity is particularly accentuated in the MBR Sister Unconditional Models (Table 3), suggesting a six-fold increase in the odds (aOR) when not accounting for familial confounding. In case of all comorbidities, the results from Model 2 and 3 (MBR Sister Unconditional, Table 3) show that adjusting for early-life factors did not affect estimates considerably, suggesting that early-life factors are not strong predictors for the association between PCOS and associated comorbidities. However, when accounting for familiar confounding (MBR Sister FE, Table 3) this association is attenuated considerably, yet still results in a four-fold increase in the odds of being diagnosed with obesity (aOR).

**Table 3:** Results from logistic regression (odds ratios and 95% confidence intervals) comparing unconditional and fixed effects logit and models predicting associations between polycystic ovary syndrome and comorbidities among women in Sweden, MBR^a^ Sister sample

	OBESITY			DEPRESSION			ANXIETY			SLEEPING, SEXUAL, EATING DISORDERS		
	**M1**	**M2**	**M3**	**M1**	**M2**	**M3**	**M1**	**M2**	**M3**	**M1**	**M2**	**M3**
**MBR Sister Unconditional**												
	***n*=77,034**			***n*=77,034**			***n*=77,034**			***n*=77,034**		
Polycystic ovary syndrome	6.091*** (5.162 - 7.186)	6.045*** (5.112 - 7.147)	5.917*** (4.979 - 7.031)	1.278** (1.026 - 1.594)	1.264** (1.014 - 1.576)	1.310** (1.049 - 1.636)	1.560*** (1.251 - 1.946)	1.561*** (1.251 - 1.948)	1.592*** (1.274 - 1.989)	1.605*** (1.166 - 2.209)	1.605*** (1.166 - 2.209)	1.658*** (1.203 - 2.284)
**MBR Sister FE**												
	***n*=4,020** **Sibling units=1,941**			***n*=7,892** **Sibling units=3,813**			***n*=6,715** **Sibling units=3,243**			***n*=3,186** **Sibling units=1,534**		
Polycystic ovary syndrome	4.242*** (2.921 - 6.159)	4.335*** (2.979 - 6.309)	4.382*** (2.981 - 6.442)	1.146 (0.820 - 1.601)	1.156 (0.827 - 1.617)	1.121 (0.793 - 1.585)	1.337 (0.934 - 1.913)	1.362* (0.950 - 1.952)	1.374* (0.951 - 1.985)	1.613* (0.954 - 2.727)	1.601* (0.942 - 2.723)	1.548 (0.906 - 2.645)

*Abbreviations*: *FE* Fixed effects, *RE* Random effect

M1: models adjusted for Birth year

M2: models adjusted for Birth year, Family background [Mother´s educational attainment, Father´s educational attainment, Mother´s origin, Father´s origin, Index women´s origin, Birth order, Mother´s age at index woman´s birth], and Early-life factors [Birth weight, One-minute Apgar score, Gestational age]

M3: models adjusted for Birth year, Family background, Early-life factors and Adult characteristics [Educational attainment, Civil status, County]

^a^MBR: Medical Birth Registry. Available for a subgroup of women born between 1973 and 1980.

^***^p<0.001

^**^p<0.01

^*^p<0.05

### Sensitivity analysis

Estimates of ORs calculated for SSE disorders separately were similar to those with the combined SSE variables (Appendix. Table A.4). When comparing the FE and random intercept models, we see that the random intercept model estimates consistently are slightly smaller in size but yields qualitatively similar results (Appendix. Table A.3.). The results of the Hausman test, which compared the fitting of the fixed and random effects models, rejected the null hypothesis in the Sister sample (Appendix. Table A.3.), confirming that the FE estimators were better fitting our data.

## Discussion

This study makes a unique contribution to the literature on comorbidities in women with PCOS by using family FE models to examine the relationship between PCOS and obesity, depression, anxiety and SSE disorders. After adjusting for a range of shared family environmental, social or genetic risk factors, we found that women with a diagnosis of PCOS have 4-fold increased odds of being diagnosed with obesity, 1.4-fold higher odds for depression or anxiety and almost 2-fold higher odds for SSE disorders, compared to a sister without PCOS.

Our finding on the strong association between PCOS and obesity is consistent with earlier epidemiological studies [13, 14, 40]. However, the direction of the association is still unclear, and most likely there are also underlying factors which affect the development of both PCOS and obesity. Similarly, a recent nationwide Swedish study on PCOS and psychiatric comorbidities, has found that women with PCOS had an 1.5-fold increased odds for having depressive and anxiety disorders [12]. These findings resonate well with our results on increased odds for depression and anxiety disorders among women with PCOS which remained robust across all models. We found an even stronger association between PCOS and SSE disorders, as much as 2.4 higher odds for having SSE as comorbidities among women with PCOS, in our Total sample. A similar pattern of results was obtained in a recent meta-analysis of 36 studies which found PCOS to be associated with an increased risk of sleeping and eating disorders and low sexual satisfaction [8].

Since previous studies have found evidence for a genetic component of PCOS based on familial clustering of the trait [41, 42], our underlying hypothesis was that there are several plausible factors at the sibling level that can impact on both PCOS and the studied comorbidities. This could be shared environmental factors, lifestyle, or a combination of genetic and environmental influences.

Even after accounting for unobserved, fixed characteristics that might influence both PCOS and the comorbidity conditions, our findings indicate that many of the previously observed associations persist, and these associations cannot be solely attributed to familial confounding. Although the sister FE approach effectively addresses shared risk factors among sisters, it does not capture variations unique to each sister. Consequently, we have made adjustments for various risk factors, available from our data, including birth year, birth order, maternal age at birth, birthweight, gestational age, one-minute Apgar score, individual educational attainment, civil status, and county of residence, aiming to account for some of the unmeasured differences in risk factors between sisters.

In our effort to minimize residual variability using observational data and focusing on full sisters who share the same biological parents, we observed that controlling for the available early-life factors had minimal impact on the previously reported coefficient estimates. This suggests that these observed early-life factors are not important confounding variables to the associations between PCOS and the examined comorbidities in our data.

### Strengths and limitations

The large, nationally representative Swedish register data allows for analyses with substantial statistical power even for relatively low-prevalence diseases in the registers such as PCOS. The high quality of the Swedish national registers with prospectively collected data [43] and a nationwide coverage also reduce the risk of selection, recall and information bias. However, this type of data source is prone to its own limitations, such as that they do not cover information on health risks or health-seeking behaviors.

Since data used for research purposes are further limited in detail in order to protect personal integrity, the research data extract do not contain the full information from the patient registers. We only had access to the more aggregated three-digit ICD-10 codes from the Swedish NPR, meaning that the E28 Ovarian dysfunction code was used throughout the analysis instead of the more detailed E28.2 Polycystic ovary syndrome code. As previously reported [12], the prevalence of PCOS is lower in the Swedish NPR than it could be expected on a population level. This could have led to measurement error. Due to information sharing between sisters, this may however be less of a concern in families where at least one sister has been diagnosed, which are the ones that are included in our FE analysis. As previously described by March et al. [3], the prevalence of PCOS is dependent on the diagnostic criteria used, finding that it can range from 8.7% up to 17.8%, depending on whether the National Institute of Health or the Rotterdam criteria were used for diagnosis. Since outpatient data is constrained by the inherent limitation of uncertainty in the diagnostic criteria used, many patients may only get diagnosed if a comorbidity condition such as obesity or sub-fertility is also present which they would primarily seek help for. This could also be the reason for the lower prevalence of PCOS in the Swedish NPR.

There was a paradigm shift in PCOS diagnosis during the follow-up period with the introduction of the Rotterdam criteria in 2003 [14] meaning that some of the index women and their sisters had been diagnosed before the shift while others were diagnosed subsequently. Additionally, outpatient specialist care was added to the Swedish NPR from 2001, and before that only inpatient care diagnoses were recorded. This could have caused a left censoring in the analysis as well as a higher concentration of more severe PCOS cases before 2001.

## Conclusion

PCOS is associated with an increased risk of obesity, depression, anxiety and eating, sleeping and sexual disorders. This association remains, net of familial confounding and observable characteristics. Early screening for comorbidities in women with PCOS and screening for PCOS in women with comorbidities is justified and early intervention may increase the quality of life in women with PCOS.

### Supplementary Information

**Supplementary Material 1.** 

## Data Availability

Swedish law prohibits the distribution of and unauthorized access to the data used for this study, stored and analyzed on a secure server managed by Statistics Sweden (https://www.scb.se/en/services/ordering-data-and-statistics/ordering-microdata/mona--statistics-swedens-platform-for-access-to-microdata/about-mona/).

## Abbreviations

aOR: Adjusted odds ratio
CI: Confidence interval
DM: Diabetes mellitus
FE: Fixed effect
ICD: International Classification of Disease
mORs: Minimally adjusted odds ratio
OSA: Obstructive sleep apnea
OD: Ovarian dysfunction
PCOS: Polycystic ovary syndrome
UREG: Register of Participation in Education
SSE: Sleeping, sexual and eating disorders
SIP: Swedish Interdisciplinary Panel
MBR: Swedish Medical Birth Register
NPR: Swedish National Patient Register
TPR: Total Population Register
