# Supplementary material for: Comorbidities in women with polycystic ovary syndrome: a sibling study
Source: BMC Womens Health. 2024 Apr 5;24:221. doi: 10.1186/s12905-024-03028-9 (PMC10996169; doi:10.1186/s12905-024-03028-9)
Supplement: Supplementary file 1 — Supplementary Material 1. [file 12905_2024_3028_MOESM1_ESM.docx]

**Appendix**

**Table A.1.** Descriptive characteristics of sub-samples

|  | WITHOUT RESTRICTION TO SISTERS  N=857,757 | CONCORDANT SISTERS: NO PCOS  N=326,422 | CONCORDANT SISTERS: PCOS  N=137 | DISCORDANT SISTERS  N=7,440 |
| --- | --- | --- | --- | --- |
| Polycystic ovary syndrome | 9,321 (1.1%) | - | 137 (100%) | 3,433 (46.1%) |
| Obesity | 24,102 (2.8%) | 8,403 (2.6%) | 24 (17.5%) | 669 (9.0%) |
| Depression | 45,488 (5.3%) | 16,739 (5.1%) | 13 (9.5%) | 512 (6.9%) |
| Anxiety | 37,655 (4.4%) | 13,551 (4.2%) | 11 (8.0%) | 455 (6.1%) |
| Sleeping, sexual and eating disorder | 14,047 (1.6%) | 5,087 (1.6%) | 6 (4.4%) | 211 (2.8%) |
| Mother´s educational attainment |  |  |  |  |
| *Primary, Secondary* | 655,220 (76.4%) | 248,404 (76.1%) | 92 (67.2%) | 5,621 (75.6%) |
| *University* | 202,537 (23.6%) | 78,018 (23.9%) | 45 (32.9%) | 1,819 (24.5%) |
| Father´s educational attainment |  |  |  |  |
| *Primary, Secondary* | 674,918 (78.7%) | 256,074 (78.5%) | 100 (72.9%) | 5,853 (78.7%) |
| *University* | 182,839 (21.3%) | 70,348 (21.6%) | 37 (27.0%) | 1,587 (21.3%) |
| Mother´s origin |  |  |  |  |
| *Sweden* | 764,336 (89.1%) | 290,642 (89.0%) | 118 (86.1%) | 6,451 (86.7%) |
| *Europe, North America and Oceania* | 80,697 (9.4%) | 30,212 (9.3%) | 16 (11.7%) | 756 (10.2%) |
| *Africa, Asia, South America* | 12,724 (1.5%) | 5,568 (1.7%) | 3 (2.2%) | 233 (3.1%) |
| Father´s origin |  |  |  |  |
| *Sweden* | 762,618 (88.9%) | 289,681 (88.7%) | 113 (82.5%) | 6,383 (85.8%) |
| *Europe, North America and Oceania* | 80,413 (9.4%) | 30,653 (9.4%) | 21 (15.3%) | 792 (10.7%) |
| *Africa, Asia, South America* | 14,726 (1.7%) | 6,088 (1.9%) | 3 (2.2%) | 265 (3.6%) |
| Origin |  |  |  |  |
| *Sweden* | 829,213 (96.7%) | 314,198 (96.3%) | 127 (92.7%) | 7,022 (94.4%) |
| *Europe, North America and Oceania* | 18,193 (2.1%) | 7,462 (2.3%) | 7 (5.1%) | 209 (2.8%) |
| *Africa, Asia, South America* | 10,351 (1.2%) | 4,762 (1.5%) | 3 (2.2%) | 209 (2.8%) |
| Birth order |  |  |  |  |
| *First born* | 381,975 (44.5%) | 118,517 (36.3%) | 52 (38.0%) | 2,720 (36.6%) |
| *Second born* | 309,822 (36.1%) | 130,497 (40.0%) | 64 (47.7%) | 2,944 (39.6%) |
| *Third born or higher* | 165,960 (19.4%) | 77,408 (23.7%) | 21 (15.3%) | 1,776 (23.9%) |
| Mother´s age at index woman´s birth, years |  |  |  |  |
| *Less than or equal to 18* | 30,071 (3.5%) | 9,508 (2.9%) | 4 (2.9%) | 210 (2.8%) |
| *Between 19-35* | 776,214 (90.5%) | 302,659 (92.7%) | 126 (92.0%) | 6,919 (93.0%) |
| *Greater than 35* | 51, 472 (6.0%) | 14,255 (4.4%) | 7 (5.1%) | 311 (4.2%) |
| Educational attainment |  |  |  |  |
| *Primary, Secondary* | 431,817 (50.3%) | 164,140 (50.3%) | 69 (50.4%) | 3,728 (50.1%) |
| *University* | 425,940 (49.7%) | 162,282 (49.7%) | 68 (49.6%) | 3,712 (49.9%) |
| Civil status |  |  |  |  |
| *Not married, not registered partnership* | 449,047 (52.4%) | 165,842 (50.8%) | 65 (47.5%) | 3,691 (49.6%) |
| *Married, registered partnership* | 408,710 (47.7%) | 160,580 (49.2%) | 72 (52.6%) | 3,749 (50.4%) |

**Table A.2** Associations between polycystic ovary syndrome and comorbidities among women in Sweden (odds ratios and 95% confidence intervals).

|  | OBESITY | | | DEPRESSION | | | ANXIETY | | | SLEEPING, SEXUAL, EATING DISORDERS | | |
| --- | --- | --- | --- | --- | --- | --- | --- | --- | --- | --- | --- | --- |
|  | **M1** | **M2** | **M3** | **M1** | **M2** | **M3** | **M1** | **M2** | **M3** | **M1** | **M2** | **M3** |
| Without restriction to sisters (n=857,757) | | | | | | | | | | | | |
| *Polycystic ovary syndrome* | 5.805***  [5.472 - 6.158] | 5.772***  [5.438- 6.126] | 5.821***  [5.492 - 6.170] | 1.652***  [1.537 - 1.775] | 1.634***  [1.521 - 1.757] | 1.688***  [1.570 - 1.815] | 1.658***  [1.535 - 1.792] | 1.639***  [1.517 - 1.771] | 1.684***  [1.557 - 1.821] | 2.361***  [2.130 - 2.618] | 2.349***  [2.119 - 2.605] | 2.372***  [2.139 - 2.631] |

*M1: models adjusted for Birth year*

*M2: models adjusted for Birth year and Family background [Mother´s educational attainment, Father´s educational attainment, Mother´s origin, Father´s origin, Index women´s origin, Birth order, Mother´s age at index woman´s birth]*

*M3: models adjusted for Birth year, Family background and Adult characteristics [Educational attainment, Civil status, County]*

****p<0.001*

***p<0.01*

**p<0.05*

**Table A.3.** Results from the sensitivity analysis. Random effects models predicting associations between polycystic ovary syndrome and comorbidities among women in Sweden, Sister sample and MBR Sister sample.

|  | OBESITY | | | DEPRESSION | | | ANXIETY | | | SLEEPING, SEXUAL, EATING DISORDERS | | |
| --- | --- | --- | --- | --- | --- | --- | --- | --- | --- | --- | --- | --- |
|  | **M1** | **M2** | **M3** | **M1** | **M2** | **M3** | **M1** | **M2** | **M3** | **M1** | **M2** | **M3** |
| Sister RE | | | | | | | | | | | | |
|  | **n=17,732**  **Sibling units=7,994** | | | **n=33,709**  **Sibling units=15,232** | | | **n=27,881**  **Sibling units=12,597** | | | **n=11,351**  **Sibling units=5,129** | | |
| *Polycystic ovary syndrome* | 4.041***  [3.313 – 4.928] | 4.041***  [3.313 - 4.930] | 4.120***  [3.373 - 5.031] | 1.392***  [1.162 - 1.668] | 1.404***  [1.171 - 1.683] | 1.405***  [1.170 - 1.688] | 1.368***  [1.131 – 1.654] | 1.382***  [1.143 - 1.672] | 1.401***  [1.156 - 1.698] | 1.975***  [1.497 - 2.607] | 1.988***  [1.506 - 2.626] | 1.965***  [1.485 - 2.599] |
| *Hausman test* | 0.239 | <0.001 | <0.001 | 0.085 | <0.001 | <0.001 | <0.001 | <0.001 | <0.001 | <0.001 | 0.069 | <0.001 |
| MBR Sister RE | | | | | | | | | | | | |
|  | **n=4,020**  **Sibling units=1,941** | | | **n=7,892**  **Sibling units=3,813** | | | **n=6,715**  **Sibling units=3,243** | | | **n=3,186**  **Sibling units=1,534** | | |
| *Polycystic ovary syndrome* | 3.795***  (2.692 - 5.350) | 3.807***  (2.696 - 5.376) | 3.875***  (2.733 - 5.496) | 1.130  (0.821 - 1.555) | 1.138  (0.826 - 1.568) | 1.132  (0.817 - 1.568) | 1.330  (0.941 - 1.879) | 1.347*  (0.953 - 1.905) | 1.397*  (0.984 - 1.982) | 1.478  (0.905 - 2.414) | 1.455  (0.888 - 2.384) | 1.507  (0.915 - 2.482) |
| Hausman test | 0.946 | 0.981 | 0.216 | 0.583 | 1.000 | 0.992 | 0.786 | 0.999 | 0.994 | 0.661 | 0.988 | 1.000 |

*Abbreviations: MBR; Medical Birth Registry, RE; random effect.*

*Hausman tests were performed between FE models from Table 2 and Table 3 and RE models from Table A.4., respectively.*

*Sister RE:*

*M1: models adjusted for Birth year*

*M2: models adjusted for Birth year and Family background [Mother´s educational attainment, Father´s educational attainment, Mother´s origin, Father´s origin, Index women´s origin, Birth order, Mother´s age at index woman´s birth]*

*M3: models adjusted for Birth year, Family and Adult characteristics [Educational attainment, Civil status, County]*

*MBR Sister RE:*

*M1: models adjusted for Birth year*

*M2: models adjusted for Birth year, Family background [Mother´s educational attainment, Father´s educational attainment, Mother´s origin, Father´s origin, Index women´s origin, Birth order, Mother´s age at index woman´s birth], and Early-life factors [Birth weight, One-minute Apgar score, Gestational age]*

*M3: models adjusted for Birth year, Family background, Early-life factors and Adult characteristics [Educational attainment, Civil status, County]*

****p<0.001*

***p<0.01*

**p<0.05*

**Table A.4.** Results from the sensitivity analysis. Associations between polycystic ovary syndrome and sleeping-, sexual-, eating disorders separately (odds ratios and 95% confidence intervals).

|  | SLEEPING DISORDERS | | | SEXUAL DISORDERS | | | EATING DISORDERS | | |
| --- | --- | --- | --- | --- | --- | --- | --- | --- | --- |
|  | **M1** | **M2** | **M3** | **M1** | **M2** | **M3** | **M1** | **M2** | **M3** |
| Without restriction to sisters (n=857,757) | | | | | | | | | |
| *Polycystic ovary syndrome* | 3.146***  [2.692 – 3.678] | 3.113***  [2.663 – 3.639] | 3.118***  [2.666 – 3.646] | 2.268***  [1.852 – 2.776] | 2.243***  [1.832 – 2.746] | 2.210***  [1.805 – 2.707] | 1.766***  [1.484 – 2.103] | 1.775***  [1.491 – 2.113] | 1.835***  [1.541 – 2.185] |

*M1: models adjusted for Birth year*

*M2: models adjusted for Birth year and Family background [Mother´s educational attainment, Father´s educational attainment, Mother´s origin, Father´s origin, Index women´s origin, Birth order, Mother´s age at index woman´s birth]*

*M3: models adjusted for Birth year, Family background and Adult characteristics [Educational attainment, Civil status]*

****p<0.001*

***p<0.01*

**p<0.05*
